# Chronic tubo-ovarian abscess complicated by hepatic portal venous gas

**DOI:** 10.1093/jscr/rjv099

**Published:** 2015-09-10

**Authors:** Sunny Onyeabor, Frederick Cason

**Affiliations:** 1 Department of Surgery, Morehouse School of Medicine, Atlanta, GA, USA; 2 General Surgery Residency Program, Morehouse School of Medicine, Atlanta, GA, USA

## Abstract

44-year-old female with massive chronic tubo-ovarian abscess complicated by hepatic portal venous gas (HPVG). She presented to the emergency department of our hospital with a diffusely tender abdomen and 2 weeks history of frequent non-bloody loose stools. She had a relevant past medical history of poorly controlled type 1 diabetes mellitus and hypertension. Abdominal computerized tomography revealed massive right abdomino-pelvic mass measuring 17.6 × 12.1 × 20 cm and diffuse HPVG. Patient underwent exploratory laparotomy, salpingo-oophorectomy, peritoneal lavage with antibiotics and treatment for septic shock. No similar case known to us has been reported in the literature previously.

## INTRODUCTION

Tubo-ovarian abscess (TOA) is a serious sequela of pelvic inflammatory disease that occurs most frequently in women of ages 20–40 but can also be seen in older or younger women [1]. Tubo-ovarian abscess is more likely to lead to complication when the ovarian mass is large or perforated. Studies have associated TOA with small bowl obstruction, hydronephrosis, septic shock, diverticulitis and intra-abdominal abscess. There is currently no literature evidence linking TOA with hepatic portal venous gas (HPVG), which occurred in our patient. Medical treatment is the main form of management for uncomplicated TOA; surgical management is reserved for complicated cases [2]. The advent of laparoscopic and interventional radiology has provided additional options for managing TOA [3]. A patient with complicated TOA may present with an acute abdomen or lower abdominal pain that may or may not radiate to the back. There might be associated history of sexually transmitted diseases (STDs), chronic use of intrauterine devices (IUDs), or no history, as in our case. Abdominal computed tomography (CT) is a good radiologic investigation for a complicated TOA, while uncomplicated TOA can be investigated using ultrasound studies. We report a case of a 44-year-old female who presented with HPVG, a rare complication of TOA, which, to our knowledge, has never previously been reported.

## CASE REPORT

**Figure 1:**
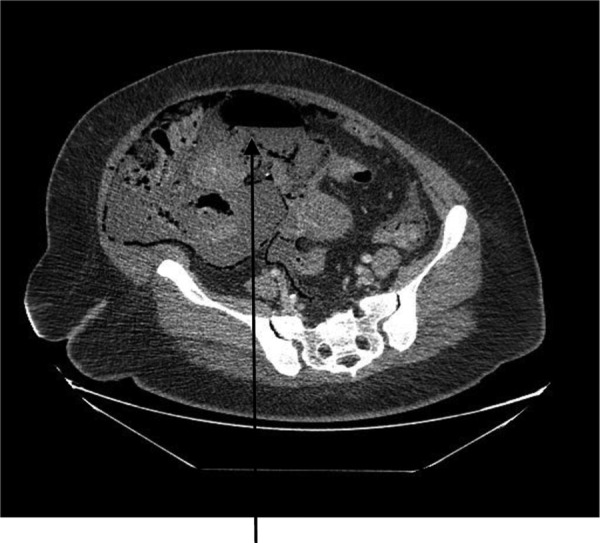
CT abdomen showing large air-filled right abdominal mass that appears to be a septic ovarian cyst.

**Figure 2:**
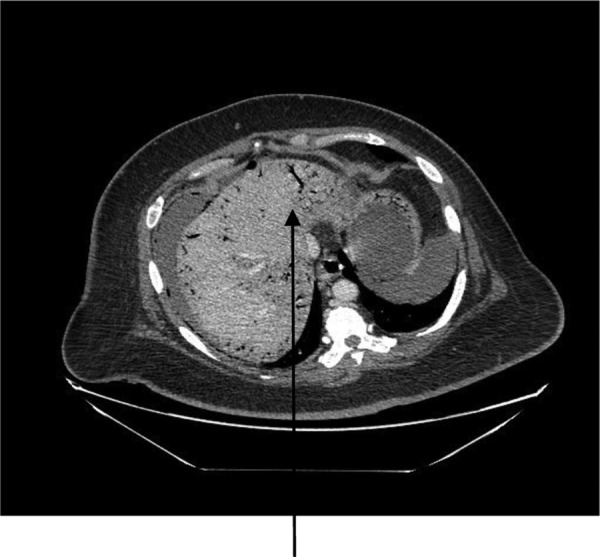
CT abdomen showing diffuse portal venous gas in the liver.

A 44-year-old female presented to the emergency department with generalized abdominal pain that became suddenly severe and diffuse 5 hours before presentation. She described the pain as sharp, alleviated by lying still on her back and worsened by moving. Pain radiated to her back. She also had watery non-bloody diarrhea that started 2 weeks before presentation. She had a medical history of poorly controlled type 1 diabetic mellitus and hypertension. No history of STDs or IUD use. Some of her labs on presentation were as follows: WBC 7.400/mm^3^, hemoglobin 15.6 g/dl and blood glucose 249 mg/dl. On examination, we found a hypotensive, dehydrated, obese female in septic shock with a tense, massively distended abdomen, which was diffusely tender. CT of the abdomen and the pelvis revealed pneumoperitoneum, a large right abdomino-pelvic mass measuring 17.6 × 12.1 × 20 cm, that appeared to be a septic ovarian cyst (Fig. 1) and diffuse portal venous gas throughout the liver (Fig. 2). Resuscitation and rehydration were initiated, and broad-spectrum intravenous antibiotics were administered. Hemodynamic instability continued, and she was emergently taken to the operating room for an exploratory laparotomy. Upon opening the abdomen pneumoperitoneum and diffuse purulent peritonitis was obvious. There was bullous emphysema of the wall of the uterus. The gallbladder had been previously removed. The stomach, duodenum, small bowel, appendix and colon were normal and involved only by serositis from the purulent peritonitis. A pus-filled right ovarian cyst, subscapular gas bubbles over the liver and a cecal bascule were the dominant abnormalities. A right salpingo-oophorectomy, appendectomy and peritoneal lavage with 15 liters saline and antibiotics were accomplished. She received a total of 7 liters of intravenous crystalloid resuscitation intraoperatively and 2 units of packed red cell transfusion. The patient was transferred to the surgical intensive care unit (SICU) after the procedure to continue resuscitation and critical care therapy for ongoing septic shock. The patient made a complete recovery and was discharged after a total of 11 days in SICU and 23 days in the hospital ward.

## DISCUSSION

Tubo-ovarian abscess is an inflammatory mass involving the fallopian tube, ovary and occasionally other adjacent pelvic organs, e.g. bowel and bladder [4]. It is usually a complication of chronic pelvic infection from prolonged IUDs use and other sources of pelvic inflammatory diseases including sexually transmitted infections. Tubo-ovarian abscess is treated medically when the abscess is small and uncomplicated. It can also be managed by percutaneous drainage when the abscess is non-ruptured. Surgical treatment is indicated for ongoing sepsis, peritonitis and other complications arising from rupture and failed medical treatment. The sequela of TOA can include intraperitoneal inflammatory adhesions to surrounding organs, episodes of pelvic and abdominal pain, infertility, and even small bowel obstruction and hydronephrosis.

Our patient presented with HPVG, which is rare (and possibly not previously reported) complication of TOA. Hepatic portal venous gas is attributed to gas-forming organisms resulting from intra-abdominal conditions such as intestinal mucosal damage or ischemia and infection in other tissues, which may be secondary to mesenteric ischemia, ulcerative colitis perforated peptic ulcer disease, gangrenous cholecystitis and ascending cholangitis; such injuries provide a portal for intraluminal gas to enter the portal venous system [5]. Our patient had no mucosal damage or evident intestinal or other gastrointestinal pathology but had diffuse peritonitis, which could have resulted in the persorption of bacteria into the mesenteric portal circulation. About 6% of HPVG patients in one report had an intra-abdominal abscess similar to what occurred in our patient [6].

Clearly, ours is a rare case of complicated TOA that required emergent surgical intervention due to ongoing septic shock, which carries a high mortality rate in the presence of HPVG [7]. Surgery is the treatment of choice for TOA with complications such as intra-abdominal abscess and HPVG [8]. There were no bowel perforations, small bowl obstruction, or signs of necrotic viscera or pancreas. Patient had no history of ischemic or inflammatory bowel diseases, or diverticulitis, pneumatosis intestinalis, peptic ulcer disease or emphysematous pyelonephritis. She had previously undergone cholecystectomy in the remote past for uncomplicated biliary colic and chronic cholecystitis. Our patient had poorly controlled type 1 diabetes mellitus (DM) and hypertension, which are similar findings in some patients who presented with HPVG from ischemic enterocolitis [9]. She also had septic shock as a result of overwhelming infection from the TOA, which could be connected to her poorly controlled DM [10]. She was treated with large-volume fluid resuscitation and intravenous antibiotics before and after surgery, which did not prevent the development of multi-organ failure and a long postoperative convalescence in the SICU and hospital ward.

## CONFLICT OF INTEREST STATEMENT

None declared.
